# Atomic and electronic structures of an extremely fragile liquid

**DOI:** 10.1038/ncomms6892

**Published:** 2014-12-18

**Authors:** Shinji Kohara, Jaakko Akola, Leonid Patrikeev, Matti Ropo, Koji Ohara, Masayoshi Itou, Akihiko Fujiwara, Jumpei Yahiro, Junpei T. Okada, Takehiko Ishikawa, Akitoshi Mizuno, Atsunobu Masuno, Yasuhiro Watanabe, Takeshi Usuki

**Affiliations:** 1 Research and Utilization Division, Japan Synchrotron Radiation Research Institute/SPring-8, 1-1-1 Kouto, Sayo-cho, Sayo, Hyogo 679-5198, Japan; 2 Schools of Materials Science, Japan Advanced Institute of Science and Technology, Nomi, Ishikawa 923-1291, Japan; 3 Department of Physics, Tampere University of Technology, PO Box 692, FI-33101 Tampere, Finland; 4 COMP Centre of Excellence, Department of Applied Physics, Aalto University, FI-00076 Aalto, Finland; 5 Peter-Grünberg-Institut PGI-1, Forschungszentrum Jülich, D-52425 Jülich, Germany; 6 Department of Mathematics and Physics, Faculty of Technology, Lappeenranta University of Technology, PO Box 20, FI-53851 Lappeenranta, Finland; 7 Department of Condensed Matter Chemistry and Physics, Graduated School of Sciences, Kyushu University, Fukuoka 812-8581, Japan; 8 Institute of Space and Astronautical Science (ISAS), Japan Aerospace Exploration Agency (JAXA), Tsukuba, Ibaraki 305-8505, Japan; 9 Department of Physics, Gakushuin University, Toshima-ku, Tokyo 171-8588, Japan; 10 Institute of Industrial Science, The University of Tokyo, Meguro-ku, Tokyo 153-8505, Japan; 11 Graduate School of Science and Engineering, Yamagata University, Yamagata 990-8560, Japan

## Abstract

The structure of high-temperature liquids is an important topic for understanding the fragility of liquids. Here we report the structure of a high-temperature non-glass-forming oxide liquid, ZrO_2_, at an atomistic and electronic level. The Bhatia–Thornton number–number structure factor of ZrO_2_ does not show a first sharp diffraction peak. The atomic structure comprises ZrO_5_, ZrO_6_ and ZrO_7_ polyhedra with a significant contribution of edge sharing of oxygen in addition to corner sharing. The variety of large oxygen coordination and polyhedral connections with short Zr–O bond lifetimes, induced by the relatively large ionic radius of zirconium, disturbs the evolution of intermediate-range ordering, which leads to a reduced electronic band gap and increased delocalization in the ionic Zr–O bonding. The details of the chemical bonding explain the extremely low viscosity of the liquid and the absence of a first sharp diffraction peak, and indicate that liquid ZrO_2_ is an extremely fragile liquid.

## Abstract (web summary)

ZrO_2_ is known to be a very poor glass former, namely an extremely fragile liquid, while its high melting temperature impedes a mechanistic understanding. Here, Kohara *et al.* show the absence of intermediate-range ordering beyond the correlation between oxide polyhedron units in ZrO_2_ upon melting.

## Graphical abstract

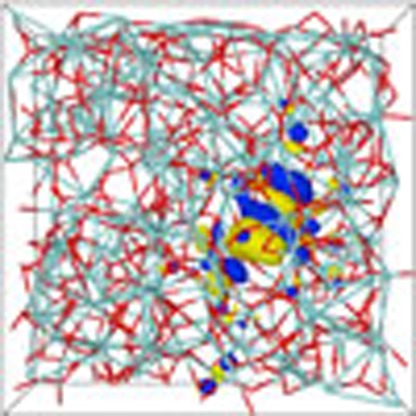

Glass formation from liquid has been studied extensively, and several theories of glass formation were established in the last century. Zachariasen^1^ and Sun^2^ proposed the basic concepts of glass formation by classifying constituents into glass formers, glass modifiers and intermediates. Furthermore, Angell^3^ introduced the concept of ‘fragility’ in glass-forming liquids (GFL) on the basis of the relationship between glass transition temperature and viscosity: liquids can be classified as ‘strong’ and ‘weak’ according to their glass-forming ability. Numerous structural studies on liquids and glasses have been performed both experimentally and theoretically^4^,^5^. The advent of advanced synchrotron/neutron sources and the development of high-performance computers have led to great progress in understanding liquid and glass structure^4^,^5^. The structural analysis of liquids with high melting points has been advanced significantly with the invention of the levitation technique^6^, especially in combination with diffraction techniques^6^. The structure of a typical non-GFL, liquid (*l*-) Al_2_O_3_ and its undercooled liquid have been studied extensively by X-ray diffraction^7^,^8^,^9^, neutron diffraction^9^,^10^ and molecular dynamics (MD) simulations^8^,^9^,^11^,^12^.

ZrO_2_ is one representative of non-glass formers and there are few reports about binary^13^ and ternary^14^ glass formation including ZrO_2_. Moreover, ZrO_2_ is commonly used as a refractory material and nucleating agent^15^ in the production of glass ceramics, suggesting that *l*-ZrO_2_ is indeed a non-GFL. As ZrO_2_ has an extremely high melting point (*T*_m_=2,715 °C), the difficulties in handling the liquid at high temperatures lead to problems in selecting suitable container materials that avoid contamination effects. We have developed a beamline levitation furnace that enables us to perform precise synchrotron X-ray diffraction measurements of liquids at extremely high temperatures.

We report here the results of precise high-energy X-ray diffraction and density measurements on containerless levitated *l*-ZrO_2_. We also carry out large-scale density functional (DF)–MD simulations, to understand the liquid properties at the atomistic and electronic level, and we compare *l*-ZrO_2_ with other non-GFLs and a typical GFL, *l*-SiO_2_. The combination of experiment and theory allows us to identify trends in single-component non-glass-forming oxide liquids, with particular focus on short- and intermediate-range ordering, the electronic band gap, maximally localized Wannier functions (WFs) of the highest valence band orbitals, and viscosity. Furthermore, we compare features of single-component non-glass-forming oxide liquids with those of other systems.

## Results

### Structure factors and real-space functions

**Figure 1:**
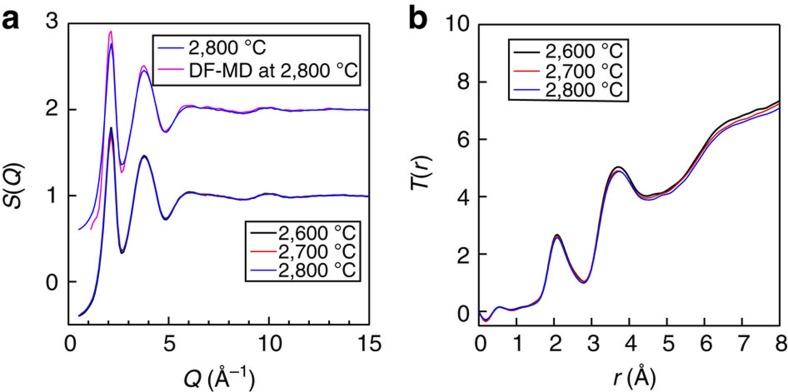
X-ray diffraction data for *l*-ZrO_2_. (**a**) Faber–Ziman total structure factors, *S*(*Q*), for *l*-ZrO_2_ at 2,600 °C–2,800 °C together with the *S*(*Q*) derived from the DF–MD simulation at 2,800 °C. Both the experimental and DF–MD simulation data at 2,800 °C are displaced upward by 1 for clarity. (**b**) Total correlation functions, *T*(*r*), for *l*-ZrO_2_ at 2,600–2,800 °C. The sharp peak observed at *Q*=2.1 Å^−1^ in the *S*(*Q*) of *l*-ZrO_2_ can be assigned to the principal peak reported by Salmon *et al.*^21^

Faber–Ziman^16^ total structure factors, *S*(*Q*), for *l*-ZrO_2_ at 2,600 °C–2,800 °C are shown in Fig. 1a. The structural change between the liquid at 2,800 °C and the undercooled liquid at 2,600 °C is very small. A sharp peak is observed at *Q*=2 Å^−1^. The total correlation functions, *T*(*r*), for *l*-ZrO_2_ (Fig. 1b) show subtle differences in real space as well. The first correlation peak observed at ~2.1 Å is assigned to Zr–O correlation and a significant tail to ~3 Å imply the formation of asymmetrical ZrO_*n*_ polyhedra in the liquid. The second peak observed at ~3.7 Å can be assigned mainly to Zr–Zr correlations and the contribution of O–O correlation is unclear due to its small weighting factor (<10%) for X-rays. The Zr–O correlation length of 2.1 Å is significantly longer than those of Si–O (~1.63 Å^17^ at 1,600 °C–2,100 °C) and Al–O (~1.78 Å^9^ at 2,127 °C) due to substantial differences in ionic radius between silicon, aluminum and zirconium ions^18^.

The observed correlation length of 2.1 Å for Zr–O agrees well with recent experimental data of Skinner *et al.*^19^ Furthermore, the increased cation–oxygen correlation length in *l*-ZrO_2_ indicates that the oxygen coordination number around zirconium is >4, because 2.1 Å is close to the sum of the ionic radii of oxygen (1.35 Å^18^) and sixfold zirconium (0.72 Å^18^). The intermediate-range structure of *l*-ZrO_2_ is then made up of large, interconnected polyhedral units and very different from those of *l*-SiO_2_ and *l*-Al_2_O_3_. This implies that the peak observed at *Q*=2 Å^−1^ in the *S*(*Q*) in Fig. 1a is not the first sharp diffraction peak (FSDP), which is typically associated with intermediate-range ordering in disordered materials, so that there is no such ordering in *l*-ZrO_2_.

**Figure 2:**
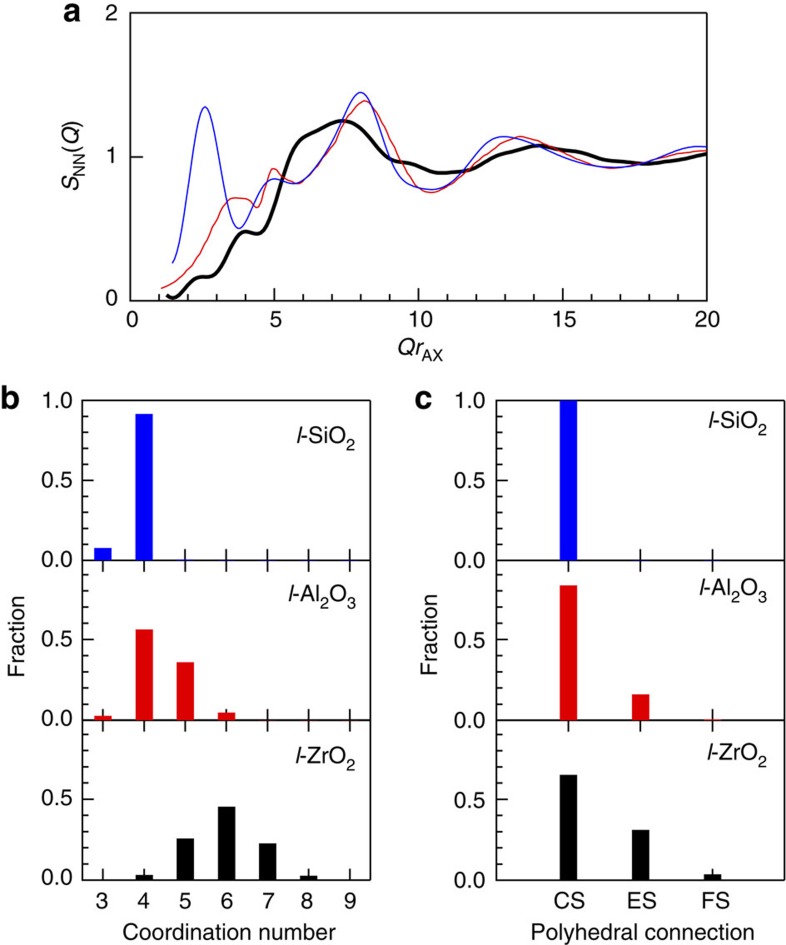
Comparison of the structural data for oxide liquids. (**a**) The Bhatia–Thornton number–number partial structure factor, *S*_NN_(*Q*), for *l*-ZrO_2_ at 2,800 °C derived from the DF–MD simulation (black curve) in comparison with those of *l*-Al_2_O_3_ at 2,127 °C (red curve)^9^ and *l*-SiO_2_ at 2,100 °C (blue curve)^22^. The momentum transfer *Q* was scaled by *r*_AX_, where *r*_AX_ is the first coordination distance between A and X in the real-space function. The Bhatia–Thornton concentration–concentration partial structure factor, *S*_CC_(*Q*), and number–concentration partial structure factor, *S*_NC_(*Q*), are shown in Supplementary Fig. 2 together with the detailed note (Supplementary Note 1). (**b**) The coordination number distribution of oxygen around the cations in *l*-ZrO_2_ at 2,800 °C, *l*-Al_2_O_3_ at 2,127 °C^9^ and *l*-SiO_2_ at 2,100 °C^22^. (**c**) The polyhedral connections in *l*-ZrO_2_ at 2,800 °C, *l*-Al_2_O_3_ at 2,127 °C^9^ and *l*-SiO_2_ at 2,100 °C^22^. CS, corner sharing of oxygen; ES, edge sharing of oxygen; FS, face sharing of oxygen.

The total structure factor, *S*(*Q*), obtained from the DF–MD simulations at 2,800 °C is shown in Fig. 1a as a magenta curve. The agreement with experimental data is excellent. Additional insight into the intermediate-range ordering of *l*-ZrO_2_, in comparison with *l*-SiO_2_ and *l*-Al_2_O_3_, can be found by calculating the Bhatia–Thornton^20^ number–number partial structure factor, *S*_NN_(*Q*),


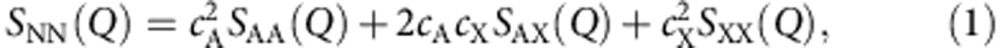


where *S*_*ij*_(*Q*) is a Faber–Ziman partial structure factor (see Supplementary Fig. 1) and *c*_*i*_ denotes the atomic fraction of chemical species *i*^21^. Figure 2a shows *S*_NN_(*Q*) (see Supplementary Fig. 2) of *l*-ZrO_2_ at 2,800 °C compared with those of *l*-Al_2_O_3_ at 2,127 °C^9^ and *l*-SiO_2_ at 2,100 °C^22^. Only *l*-SiO_2_ exhibits FSDP at *Qr*_AX_=2.7 (*r*_AX_ is the atomic cation (A)–anion (X) distance in AX polyhedra to normalize *Q*). Neither *l*-Al_2_O_3_ nor *l*-ZrO_2_ show an FSDP in the *S*_NN_(*Q*), whereas it is present in the total X-ray and neutron *S*(*Q*) of *l*-Al_2_O_3_ (see Supplementary Fig. 3)^9^ due to the contribution of weighting factors for X-rays and neutrons. As Bhatia–Thornton *S*_NN_(*Q*) can eliminate the weighting factors, the absence of FSDP in the *S*_NN_(*Q*) of *l*-ZrO_2_ is a signature of non-glass-forming behaviour.

The partial pair correlation functions, *g*_*ij*_(*r*), of *l*-ZrO_2_ derived from the DF–MD simulations are presented in Supplementary Fig. 4, together with those of the high-temperature phase of crystalline (*c*-) ZrO_2_ (ref. 23). The first correlation peak of *g*_ZrO_(*r*) for *l*-ZrO_2_ is broad and shows a tail up to 2.8 Å. A very broad first maximum in *g*_OO_(*r*) overlaps the first correlation peaks of *g*_ZrO_(*r*) and *g*_ZrZr_(*r*), indicating that the oxygen coordination is very different from those of *l*-SiO_2_ and *l*-Al_2_O_3_, where corner-sharing tetrahedra are predominant.

### Analysis of three-dimensional atomic arrangement

The average coordination number of oxygen around zirconium, *N*_ZrO_, calculated up to 2.8 Å for *l*-ZrO_2_ is 5.9 for the DF–MD configuration (*N*_OZr_~3), significantly lower than 8 in *c*-ZrO_2_. Recently, Skinner *et al.*^19^ reported that *N*_ZrO_=6.1 at 2,897 °C, which is close to our value (5.9 at 2,800 °C), although the estimated density in ref. 19 is smaller than our experimentally measured value. The oxygen coordination number around zirconium is significantly larger than *N*_Si-O_=3.9 in *l*-SiO_2_ at 2,100 °C^22^ and *N*_Al-O_=4.4 in *l*-Al_2_O_3_ at 2,127 °C^9^, due to large differences between the ionic radii of Si, Al and Zr. Similarly, large oxygen numbers reported on other non-GFLs (*N*_Y-O_=5.5 in *l*-Y_2_O_3_ at 2,597 °C^19^ and *N*_La-O_=5.9 in *l*-La_2_O_3_ at 2,497 °C^19^) support our argument that a large oxygen coordination in *l*-ZrO_2_ is a signature of non-GFLs and is associated with the absence of FSDP.

The coordination number distributions of *l*-SiO_2_ (ref. 22), *l*-Al_2_O_3_ (ref. 9) and *l*-ZrO_2_ calculated from the structural models are shown in Fig. 2b. SiO_4_ tetrahedra are predominant in *l*-SiO_2_ (ref. 22), while *l*-Al_2_O_3_ comprises AlO_3_, AlO_5_ and AlO_6_ units, as well as fourfold Al. For *l*-ZrO_2_, the most common configurations are ZrO_5_, ZrO_6_ and ZrO_7_. Although ZrO_2_ and Al_2_O_3_ have different stoichiometries, this comparison supports our view that the variety of oxygen coordination around cations in *l*-ZrO_2_ is another characteristic feature of non-glass-forming behaviour, because it can disturb the evolution of intermediate-range ordering.

To obtain structural features beyond the first coordination distance, a polyhedral connection analysis for *l*-SiO_2_ (ref. 22), *l*-Al_2_O_3_ (ref. 9) and *l*-ZrO_2_ has been carried out. Figure 2c shows the fraction of corner sharing, edge sharing and face sharing of polyhedral units in the liquids. Corner sharing of oxygen is prevalent in *l*-SiO_2_ (ref. 22), which is a unique feature of GFLs according to Zachariasen^1^. However, both *l*-Al_2_O_3_ and *l*-ZrO_2_ exhibit a considerable oxygen edge sharing, so that the variety of polyhedral connections is a further characteristic feature of single-component, non-glass-forming oxide liquids.

**Figure 3:**
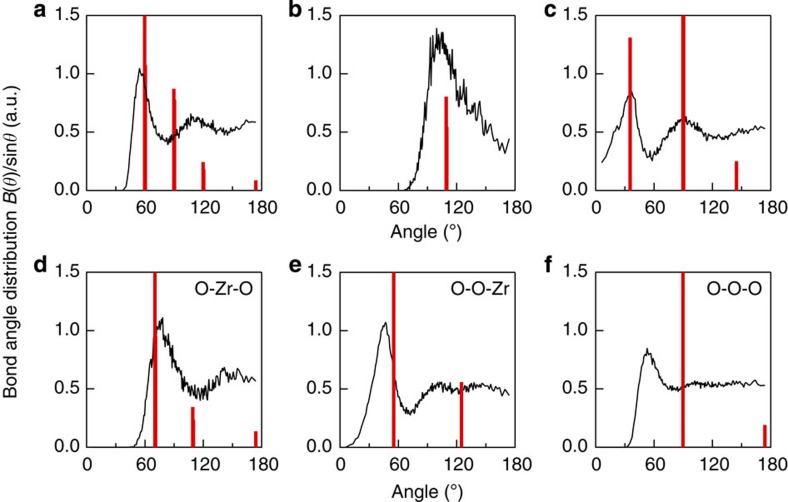
Bond angle distributions for *l*-ZrO_2_ at 2,800 °C and *c*-ZrO_2_. (**a**) The bond angle configurations Zr–Zr–Zr, (**b**) Zr–O–Zr, (**c**) Zr–Zr–O, (**d**) O–Zr–O, (**e**) O–O–Zr and (**f**) O–O–O. The *B*(*θ*)/sin*θ* data for *c*-ZrO_2_ has been scaled by a factor of 20 for clarity.

The bond angle distributions for *l*-ZrO_2_ at 2,800 °C derived from the DF–MD simulations are shown in Fig. 3. The peak at ~105° in the Zr–O–Zr distribution is very different from that at ~145° in the Si–O–Si distribution of *l*-SiO_2_ (ref. 22), but similar to the Al–O–Al distribution in *l*-Al_2_O_3_ (ref. 9). The O–Zr–O distribution shows a principal peak at 75° and a small peak at 150°. They are very different from the angle of a typical AX_4_ tetrahedron (109.47°), but similar to the O–Al–O distribution in *l*-Al_2_O_3_ (ref. 9). This indicates that the wide variation of ZrO_*n*_ polyhedra (ZrO_5_, ZrO_6_ and ZrO_7_) is another characteristic feature of *l*-ZrO_2_. The distributions of Zr–Zr–Zr, Zr–Zr–O and O–O–Zr are also similar to those of *l*-Al_2_O_3_ (ref. 9). The O–O–O distribution of *l*-ZrO_2_ shows a maximum only at 60°, while the O–O–O distribution of *l*-Al_2_O_3_ (ref. 9) has a prominent peak at 60° and a small but distinct peak at ~110° (signature of an anion in tetrahedral coordination). However, *l*-ZrO_2_ does not have this feature, implying that its oxygen coordination is different.

The bond angle distributions for the high-temperature phase of *c*-ZrO_2_ (ref. 23) are shown in Fig. 3 as red lines. Although there are similarities in the liquid and crystal, the most striking difference is in the O–O–O distribution: *l*-ZrO_2_ has a prominent peak at 60°, while *c*-ZrO_2_ shows intense peaks at 90° and 174°. This difference is caused by the variation of short-range ordering in the ZrO_*n*_ polyhedral units.

### Analysis of electronic structure

**Figure 4:**
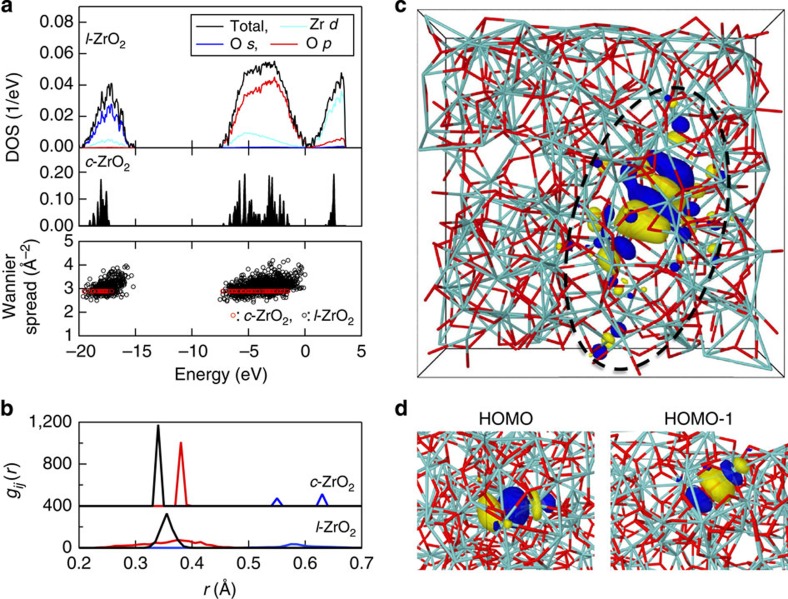
The electronic structure of *c*-ZrO_2_ and *l*-ZrO_2_. (**a**) DOS and its projections onto atomic orbitals for the higher valence bands and conduction band, together with the Wannier function spreads (occupied states). (**b**) The partial pair correlation functions, *g*_*ij*_(*r*), of WF centres, Zr–*C*_w_ (black), O–*C*_w_ (red) and *C*_w_–*C*_w_ (blue). (**c**) A visualization of the HOMO state (KS orbital) in *l*-ZrO_2_. (**d**) Two WFs corresponding to HOMO and HOMO-1 of *l*-ZrO_2_. Zr and O atoms are shown in cyan and red colour, respectively. Yellow and blue isosurfaces denote different signs of the wavefunction nodes.

The electronic structure analysis was performed in terms of the electronic density of states (DOS), WFs and effective charges for snapshots of the high-temperature phases of *c*-ZrO_2_ and *l*-ZrO_2_. The DOS (above −20 eV) of *c*- and *l*-ZrO_2_, and its projections (P-DOS) for *l*-ZrO_2_ are shown in Fig. 4a. The P-DOS plots reveal that this part of the electronic spectrum is associated mainly with oxygen (O-2*p* orbitals) and the Zr semicore states corresponding to the atomic Zr-4*s* and Zr-4*p* orbitals are deeper (below −20 eV, not shown). The zirconium *d*-component dominates in the conduction band of *l*-ZrO_2_. The effect of high temperature on the distorted ZrO_*n*_ polyhedra in *l*-ZrO_2_ is evident as a broadening of the energy bands and the gap between the valence and conduction bands has disappeared (the calculated band gap is 3.26 eV for *c*-ZrO_2_).

The difference between the electronegativities of Zr (1.3) and O (3.5) indicates that the chemical bonding between the two elements is mainly ionic and this is supported by the significant weight on oxygen P-DOS for the highest valence band. Table 1 summarizes the atomic charges and volumes of Zr cations and O anions in *c*- and *l*-ZrO_2_ obtained by the Bader method^24^. For *l*-ZrO_2_, the evaluated effective charges are +2.62*e* and −1.31*e* for Zr and O, respectively, and reflect the ionic bonding. The atomic charges in *l*-ZrO_2_ are very similar to those in the crystalline phase, which is in accordance with our previous studies on MgO–SiO_2_ (ref. 25) and CaO–Al_2_O_3_ (ref. 26) glasses. The associated atomic volumes imply that the increased oxygen volume in *l*-ZrO_2_ compensates for the decreased oxygen coordination, and this results in comparable atomic charges for the two phases. Similar behaviour has been found for MgO–SiO_2_ glass^25^.

The maximally localized WF can be considered as the natural generalization of ‘localized molecular orbitals’ in solids and they provide valuable insight into chemical bonding^27^,^28^. The WFs have been obtained from the occupied Kohn–Sham (KS) orbitals by a unitary transformation where the spatial extension (spread) of the WF orbitals is minimized. For each WF orbital, the Wannier centre (*C*_w_) location denotes the most probable point for locating an electron (or electron pair in case of a spin-degenerate orbital) and the corresponding Wannier spread is a direct measure of the degree of localization. The distribution of Wannier spreads and *C*_w_s in terms of pair correlation functions are shown in Fig. 4a,b, respectively. The *g*_*ij*_(*r*) for Zr–*C*_w_, O–*C*_w_ and *C*_w_–*C*_w_ (Fig. 4b) have maxima well below 1 Å, showing that *C*_w_s are close to Zr and O atoms. The ionic character of chemical bonding is clearly visible along Zr–O bonds, where the associated WF centres (electron pairs from higher valence bands) are always close to oxygen and there are four *C*_w_s around each O. In the high-temperature phase of *c*-ZrO_2_, the corresponding *C*_w_s are symmetrically aligned along Zr–O bonds, as O is tetrahedrally coordinated by Zr (Fig. 4b, O–*C*_w_ partial). The oxygen coordination is smaller (*N*_OZr_~3) and less regular in the liquid, and there is considerable scatter in both the *C*_w_ locations and spreads. The latter show (Fig. 4a, bottom panel) that WFs are considerably less localized than the crystalline reference value of ~2.9.

The highest occupied molecular orbital (HOMO) has been also visualized in Fig. 4c, where the KS orbital (HOMO) is delocalized over a group of atoms highlighted by a dashed circle, while the transformed WFs for HOMO and HOMO-1 (‘molecular orbitals’, Fig. 4d) are each localized over one Zr–O bond. The WF shapes of these examples are very similar to those in the high-temperature phase of *c*-ZrO_2_, but there are also cases with considerable deviation, as can be expected from the scatter of WF spreads (Fig. 4a).

## Discussion

**Figure 5:**
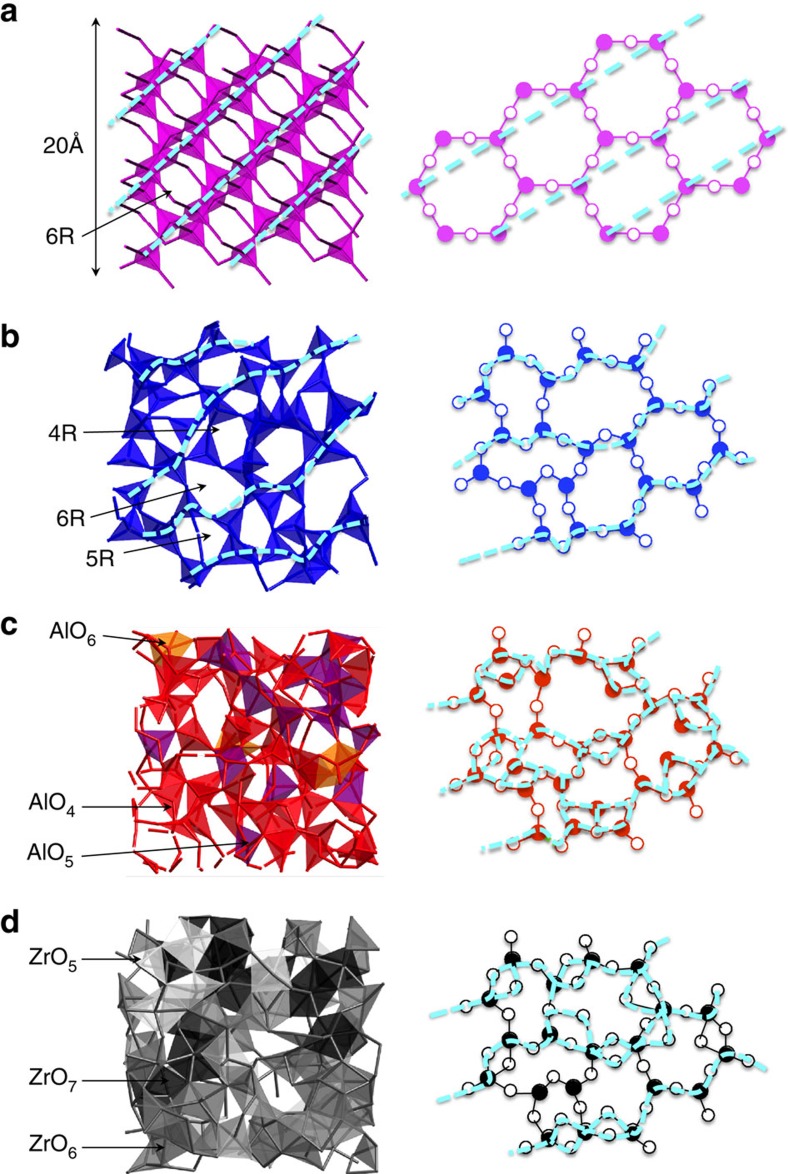
The atomic structure of oxide liquids. (**a**) *c*-SiO_2_ (for reference), (**b**) *l*-SiO_2_, (**c**) *l*-Al_2_O_3_ and (**d**) *l*-ZrO_2_. Colour code (right panels): Si, magenta and blue spheres; Al, red; Zr, black; and O, white. The periodicity of cage boundaries is highlighted by cyan dashed lines and curves.

The origin of FSDP associated with the formation of intermediate-range ordering in oxide glasses and liquids remains controversial, because the inherent disorder complicates the ability of AX polyhedral connections to form an A–X network. SiO_2_ has an exceptionally high glass-forming ability and the origin of FSDP in SiO_2_ has often been studied. The results are summarized in ref. 29. The random network model of Zachariasen^1^ and modified for an oxide glass modified in refs 30, 31 (illustrated in Fig. 7 of ref. 29) suggests that the intermediate-range ordering arises from the periodicity of boundaries between successive small cages in the network formed by connected, regular SiO_4_ tetrahedra with shared oxygen atoms at the corners. It has also been demonstrated that small cages are topologically disordered^32^, resulting in a broad distribution of ring sizes from 3-fold to 12-fold rings centred at 6-fold rings^25^,^33^. This is reflected in the *S*_NN_(*Q*) of *l*-SiO_2_ (Fig. 2a), where the FSDP width is broader than that of the corresponding Bragg peak in the crystalline phase (β-cristobalite, *c*-SiO_2_), where only a sixfold ring donates. Figure 5a,b show three-dimensional atomic configurations and schematic illustrations for *c*-SiO_2_ and *l*-SiO_2_, respectively. The crystalline phase exhibits only sixfold rings of six SiO_4_ tetrahedra, yielding a long-range periodicity (dashed cyan lines in Fig. 5a). However, some pseudo Bragg planes (dashed cyan lines in the left panel of Fig. 5b) can be recognized in *l*-SiO_2_. Although the introduction of different ring sizes can easily modify the crystalline topological order (Fig. 5b), the interconnection of regular SiO_4_ tetrahedra with shared oxygen at corners only yields the broadened Bragg peak as FSDP.

In *l*-Al_2_O_3_, a considerable fraction of AlO_5_ units associated with the formation of OAl_3_ triclusters^34^ and the contribution of edge-sharing atoms (see Fig. 5c)^9^ are necessary to compensate the negative charge of AlO_4_, because the nominal charge of the Al cation is three. We suggest that the variety of oxygen coordination (see Fig. 2b) and polyhedral sharing (Fig. 2c) disturb the formation of intermediate-range ordering in *l*-Al_2_O_3_. This is apparent in the absence of FSDP in the *S*_NN_(*Q*) of *l*-Al_2_O_3_ in Fig. 2a, despite the similarity between *l*-SiO_2_ and *l*-Al_2_O_3_ due to the predominant AlO_4_ units and corner sharing of oxygen in *l*-Al_2_O_3_ (note that the stoichiometry of Al_2_O_3_ is different from that of SiO_2_). The three-dimensional atomic configuration and schematic illustration of *l*-Al_2_O_3_ are illustrated in Fig. 5c, where the periodicity of boundaries is less obvious, due to the large contribution of AlO_5_ (purple polyhedra) and edge sharing of oxygen.

As can be seen in Fig. 2a, an FSDP is absent in the *S*_NN_(*Q*) of *l*-ZrO_2_, because the variety of short-range structural units with large oxygen coordination, ZrO_5_, ZrO_6_ and ZrO_7_, and the large contribution of oxygen edge sharing prevents the formation of intermediate-range ordering. A similar feature can be expected in *l*-Y_2_O_3_ and *l*-La_2_O_3_, because their Faber–Ziman partial structure factors, *S*_*ij*_(*Q*), do not contribute to the expected *Q* position of~1 Å^−1^ for a FSDP^19^. Short-range structural disordering in *l*-ZrO_2_ is further demonstrated in the three-dimensional atomic configuration and the schematic illustration of *l*-ZrO_2_ (Fig. 5d). The periodicity of boundaries (dashed cyan lines) is suppressed by the formation of edge-sharing of oxygen associated with the formation of ZrO_5_ (white polyhedra), ZrO_6_ (grey polyhedra) and ZrO_7_ (black polyhedra). Although ZrO_2_ forms a network structure by interconnecting AX polyhedra in the liquid phase, we have shown that the various short-range structural units and their connectivity cause disorder at the intermediate range and prevent the evolution of a FSDP in the liquid. Our results demonstrate that the absence of FSDP in *S*_NN_(*Q*) can be an important indicator for single-component non-glass-forming oxide liquids, but it does not necessarily apply similarly to other non-GFLs.

Although ZrO_2_ and Al_2_O_3_ have different stoichiometries, the absence of FSDP in the *S*_NN_(*Q*) suggests that they are both very ‘fragile’ liquids^3^. This suggestion is supported by a comparison with *l*-ZnCl_2_, which is recognized as an intermediate case between a ‘strong’ and ‘fragile’ liquid. *l*-ZnCl_2_ shows a well-defined, but not sharp, FSDP in the *S*_NN_(*Q*) by the contribution of corner sharing of ZnCl_4_ tetrahedra, while edge sharing is also found^35^. This behaviour of ‘fragile’ glass former is very similar to *l*-GeSe_2_ where the FSDP in *S*_NN_(*Q*) is weak, and a considerable fraction of edge sharing of GeSe_4_ tetrahedra contribute^36^, as in glassy (*g*-) GeSe_2_ (ref. 37). We suggest that the liquid fragility increases with the contribution of edge sharing of tetrahedra, as discussed in ref. 38. However, the typical glass former *g*-GeO_2_ shows the contribution of only corner sharing of GeO_4_ tetrahedra^33^ that results in a sharp FSDP, while *g*-Ge-Te systems do not exhibit FSDP due to co-existing octahedral and tetrahedral Ge–Te polyhedra. Here the Te–Ge–Te bond angle distribution is peaked around 90°, quite different from 109.47° of regular tetrahedra in *g*-GeO_2_ (refs 39, 40, 41). We conclude that the magnitude of the FSDP is sensitive to the variety in atomic coordination and polyhedral connections, which are connected in turn to the difference in ionic radii between constituent anions and cations.

The fragility of *l*-ZrO_2_ is confirmed by comparing the thermodynamic parameters of *l*-ZrO_2_ and *l*-Al_2_O_3_. Recent MD simulation for *l*-Al_2_O_3_ at 2,227 °C^42^ reported a zero-frequency viscosity of 2.5 × 10^−2^ Pa s^−1^, while the viscosity of *l*-SiO_2_ at 1,652 °C (a typical ‘strong’ liquid^3^) is 6.12 × 10^6^ Pa s^−1^ (ref. 43). The zero-frequency viscosity value of *l*-Al_2_O_3_ is comparable to the results of the recent inelastic X-ray scattering measurement^44^ and the macroscopic shear viscosity of 3.3 × 10^−2^ Pa s^−1^ at 2,213 °C^45^, confirming that it is a ‘fragile’ liquid. The self-diffusion coefficients for Zr and O in *l*-ZrO_2_ derived from our DF–MD simulations are 3.6 × 10^−5^  and 7.1 × 10^−5^ cm^2^ s^−1^, respectively, at 2,800 °C. The viscosity of *l*-ZrO_2_, estimated by assuming spherical particles and applying the Stokes–Einstein equation, is ~2 × 10^−3^ Pa s^−1^ at 2,800 °C, indicating that *l*-ZrO_2_ is an extremely fragile liquid. This conclusion is further supported by the Zr–O bond lifetime analysis of DF–MD simulations (Supplementary Note 2), which shows that 50% of the bonds break within 185 fs at 2,800 °C (Supplementary Fig. 5). The Zr–O bond lifetime is extremely short when it is compared with the observation that the exchange-rate between bridging and non-bridging oxygen atoms in a silicate melt is within a nanosecond-to-microsecond time scale^46^. This behaviour of Zr–O bonds is closely related to the variety of ZrO_*n*_ polyhedral units and polyhedral connections with a reduced electronic band gap and increased delocalization in the ionic Zr–O bonding.

We study the atomic and electronic structure of non-glass-forming *l*-ZrO_2_ with an extremely high melting point by using a combination of containerless processing, synchrotron X-ray diffraction, density measurements and DF–MD simulations. Although a sharp peak is observed in the X-ray total structure factors, we find that FSDP is absent in the Bhatia–Thornton *S*_NN_(*Q*). We show that the variety of short-range structural units with large oxygen coordination and their associated distortion due to edge sharing are signatures of single-component non-glass-forming oxide liquids. The absence of FSDP is ascribed to the variety of ZrO_*n*_ polyhedral units induced by the large ionic radius of Zr cation. This is reflected in the short lifetime of Zr–O bonds (and polyhedral units), which prevents the evolution of intermediate-range ordering. These structural features are coupled to irregularity and reduced localization in the ionic Zr–O bonds with short lifetime, yielding a reduced electronic band gap in *l*-ZrO_2_ and a low viscosity of the liquid. By comparing our results for *l*-ZrO_2_ with other GFLs, non-GFLs and glasses, we conclude that the absence of FSDP in the *S*_NN_(*Q*), associated with a short lifetime of Zr–O bonds and extremely low viscosity, is a feature of single-component non-glass-forming oxide liquids, although this does not necessarily apply to all non-GFLs. The DF–MD simulation results support the observed absence of FSDP and suggest that *l*-ZrO_2_ is an extremely fragile liquid. Finally, the containerless preparation and measurement techniques open up fresh capabilities to study new features in extremely high-temperature liquids, and we demonstrate the importance of combining experiment and theory to understand the nature of liquids at the atomistic (structure and dynamics) and electronic (chemical bonding) level.

## Methods

### Density measurement

The density measurement of *l*-ZrO_2_ was performed with an aerodynamic levitator^6^,^47^,^48^. A small ZrO_2_ sample whose diameter was around 2 mm was set in a shallow nozzle where the sample was aerodynamically levitated. The levitated sample was then heated by a 100-W CO_2_ laser and a 500-W Nd:YAG laser. The temperature of the sample was measured by a single colour pyrometer. The weight of the recovered sample was measured. The temperature was calibrated using the given melting temperature (*T*_m_=2,715 °C) in density measurements. The details of measurement can be found in the Supplementary Note 3 and typical image of the levitated specimen and the density data is shown in Supplementary Figs 6 and 7, respectively.

### High-energy X-ray diffraction measurement

The high-energy X-ray diffraction experiments of *l*-ZrO_2_ were carried out at the BL04B2 and the BL08W beamlines^49^ at the SPring-8 synchrotron radiation facility, using the aerodynamic levitation technique^6^,^47^,^48^,^49^. The energy of the incident X-rays was 113 keV (BL04B2) and 116 keV (BL08W). The ZrO_2_ sample of 2-mm size was levitated by dry air and heated by a 100-W CO_2_ laser. The temperature of the sample was monitored by a two-colour pyrometer. As can be seen in Supplementary Fig. 8, the background of the instrument was successfully reduced due to adequate shielding of the detector and the optimization of beam stop. The measured X-ray diffraction data were corrected for polarization, absorption and background, and the contribution of Compton scattering was subtracted using standard analysis procedures^49^. The corrected data sets were normalized to give the Faber–Ziman^16^ total structure factor *S*(*Q*) and the total correlation function *T*(*r*) was obtained by a Fourier transformation of *S*(*Q*).

### DF–MD simulation

The combined DF and MD simulations were performed with the CP2K programme package ( http://www.cp2k.org)^50^. The CP2K method uses two representations for the KS orbitals and electron density: localized Gaussian and plane wave basis sets. For the Gaussian-based (localized) expansion of the KS orbitals, we used a library of contracted molecularly optimized valence double-zeta plus polarization basis sets^51^ and the complementary plane wave basis set for electron density has a cutoff of 400 Ry. The generalized gradient approximation of Perdew, Burke and Ernzerhof^52^ (PBE) was adopted for the exchange-correlation energy functional and the valence electron–ion interaction was based on the norm-conserving and separable pseudopotentials of the form derived by Goedecker *et al.*^53^ We consider the following valence configurations: Zr (Cl4s^2^4p^2^5s^2^5d^2^) and O (2s^2^2p^4^). Periodic boundary conditions were used, with a single point (*k*=0) in the Brillouin zone. Effective charges of individual atoms were evaluated from electron density by integrating electronic charge inside the corresponding atomic volumes^24^. For reference, electronic structure of the high-temperature phase of *c*-ZrO_2_ (*T*>2,370 °C) is computed for a sample of 324 atoms.

The initial atomic configuration is created by a reverse Monte Carlo (RMC) simulation with high-energy X-ray diffraction data on 501 atoms in a cubic box of 18.98 Å (experimental density, 0.0733 atoms per Å^3^). The RMC++ programme code^54^ was used. The Born–Oppenheimer MD simulations were performed with a Nóse–Hoover thermostat^55^ and time steps of 2 fs (initialization) and 1 fs (data collecting). The system was simulated at 3,100 K (~2,800 °C) for a total of 30 ps, where the last 10 ps were used for data collection^55^. The corresponding mean-square displacement of atoms shows clearly a liquid behaviour (diffusion). The comparison of the partial pair correlation functions, *g*_*ij*_(*r*), between the initial RMC configuration (start) and the DF–MD simulation is shown in Supplementary Fig. 9. The sharp shape of O–O *g*_ij_(*r*) in the RMC model is artificially sharp due to small weighting factor for X-rays, while the shape of O–O *g*_ij_(*r*) is reasonable in the DF–MD simulations. The system lost its memory of the initial (RMC) starting structure within a few picoseconds (Zr–O bond lifetimes ~185 fs).

## Author contributions

S.K. and J.A. designed the research. S.K., K.O., M.I., J.Y., J.T.O., T.I, A. Mizuno, A. Masuno, Y.W. and T.U. performed experiments. J.A. and L.P. carried out DF–MD simulations. S.K., J.A., L.P., M.R. and A.F. analysed the data. S.K., J.A. and A.F. wrote the paper.

## Additional information

**How to cite this article**: Kohara, S. *et al.* Atomic and electronic structures of an extremely fragile liquid. *Nat. Commun.* 5:5892 doi: 10.1038/ncomms6892 (2014).

## Supplementary Material

Supplementary Information Supplementary Figures 1-9, Supplementary Table 1, Supplementary Notes 1-3 and Supplementary References

## Figures and Tables

**Table 1:** Atomic charges and volumes obtained by the Bader method.

	**Zr**		**O**	
	***Q***_**eff**_**(*****e*****)**	***V***_**at**_**(Å**^3^**)**	***Q***_**eff**_**(*****e*****)**	***V***_**at**_**(Å**^3^**)**
*c*-ZrO_2_	2.60	10.54	−1.30	11.65
*l*-ZrO_2_	2.62	11.48	−1.31	14.72

Comparison between the results obtained by the Bader and Voronoi methods are listed in Supplementary Table 1.
