# Supplementary material for: Atomic and electronic structures of an extremely fragile liquid
Source: Nat Commun. 2014 Dec 18;5:5892. doi: 10.1038/ncomms6892 (PMC4284809; doi:10.1038/ncomms6892)
Supplement: Supplementary Information — Supplementary Figures 1-9, Supplementary Table 1, Supplementary Notes 1-3 and Supplementary References [file ncomms6892-s1.pdf]

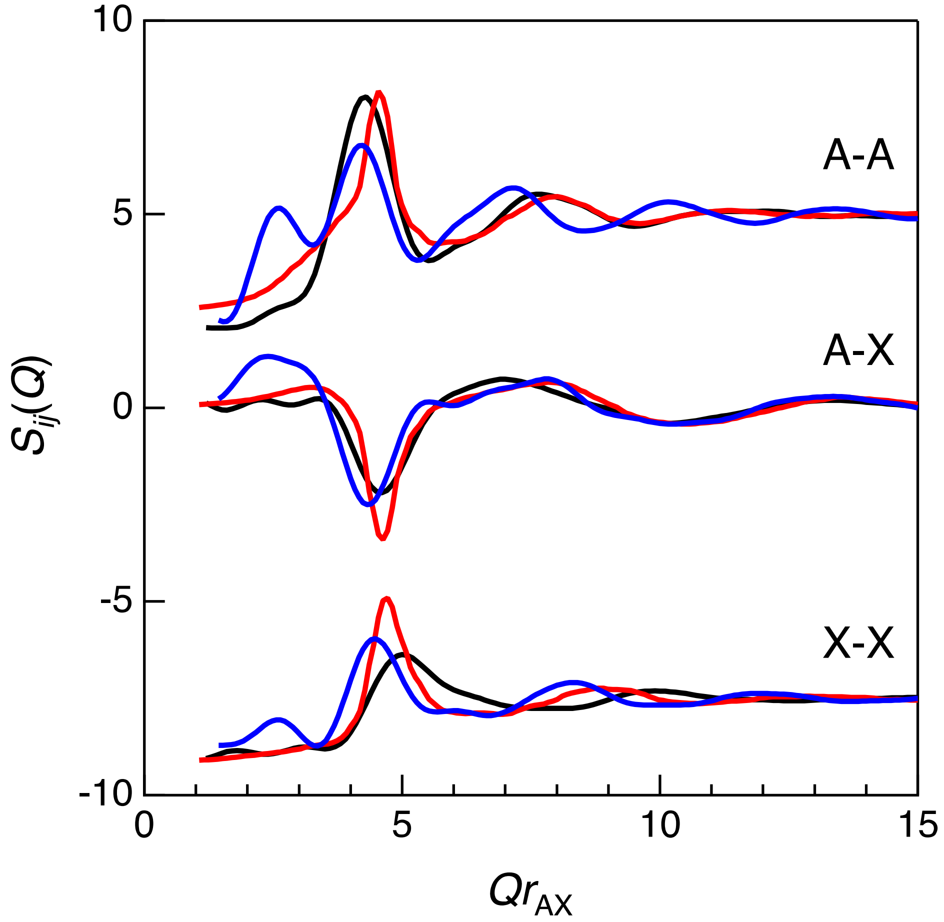

Supplementary Figure 1 | Faber-Ziman partial structure factors, $S_{ij}(Q)$, for liquid (*l*)-ZrO_2 at 2800°C (black), *l*-Al_2O_3 at 2127°C (red)[1], and *l*-SiO_2 at 2100°C (blue)[2]. The momentum transfer Q was scaled by r_{AX},where r_{AX} is the first coordination distance in the real-space function.

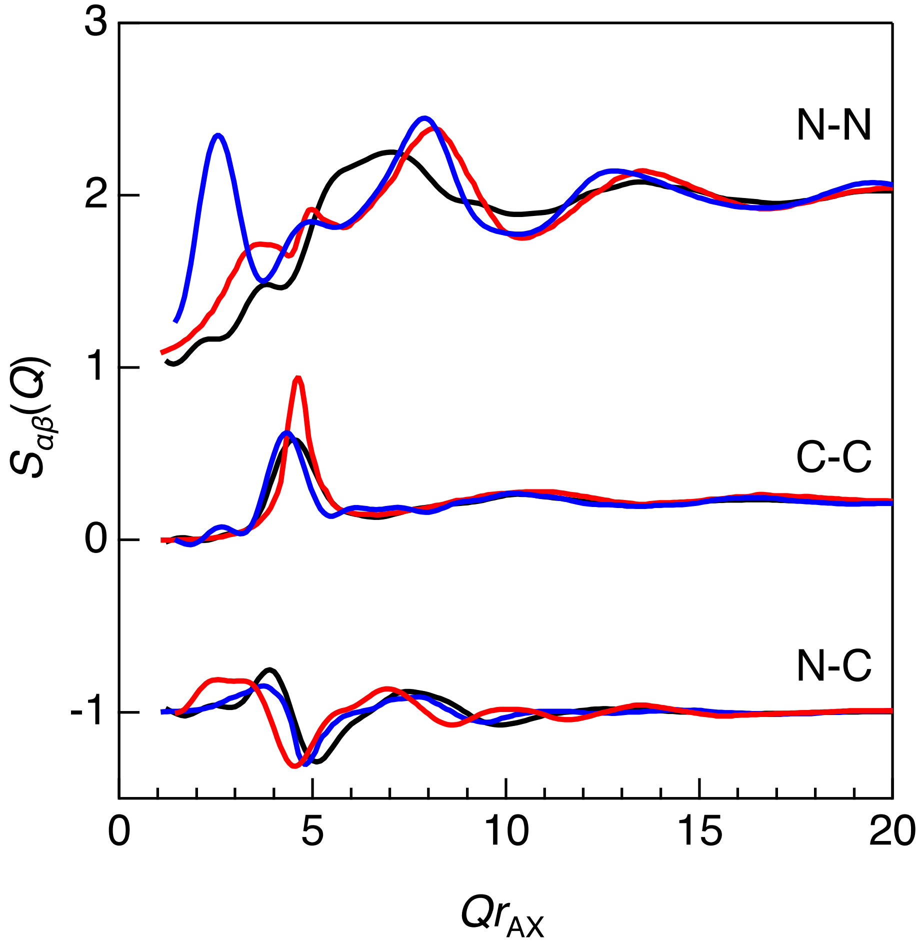

Supplementary Figure 2 | Bhatia-Thornton partial structure factors, $S_{\alpha\beta}(Q)$, for *l*-ZrO_2 at 2800°C derived from the density functional (DF)-molecular dynamics (MD) simulation (black curves) compared with those of *l*-Al_2O_3 at 2127°C (red curves)[1] and *l*-SiO_2 at 2100 °C (blue curves)[2]. The momentum transfer Q was scaled by r_{AX}.

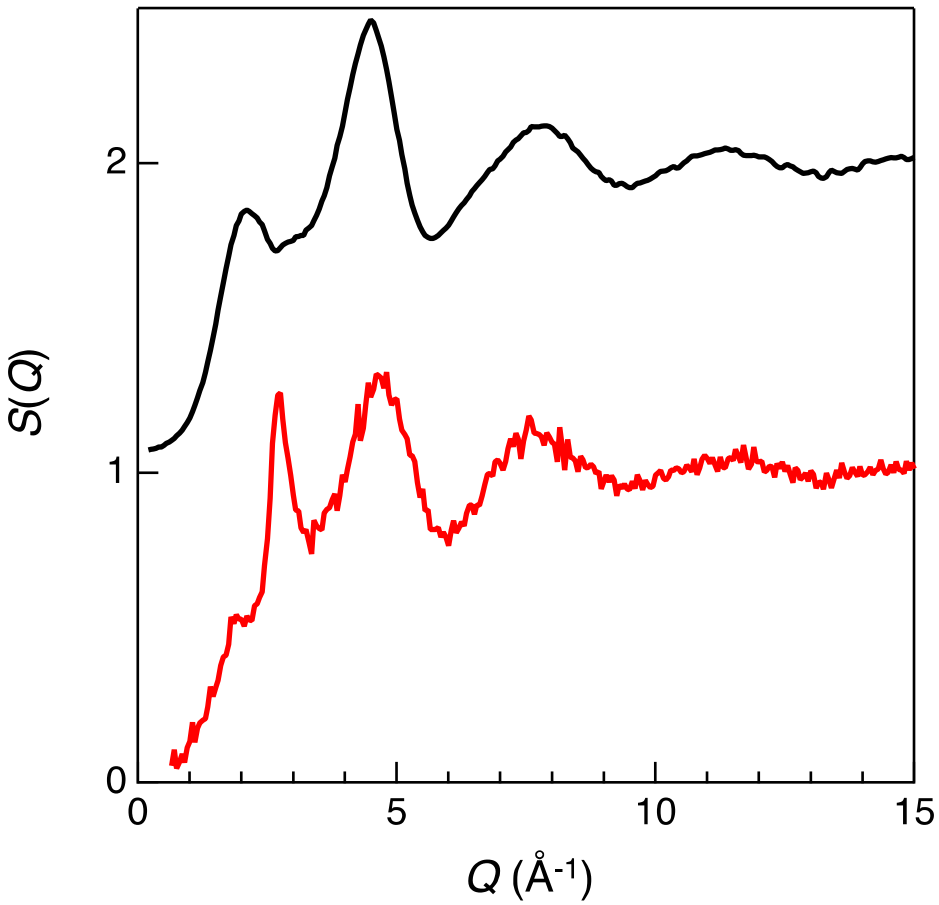

Supplementary Figure 3 | X-ray (black) and neutron (red) structure factors, *S*(*Q*), for *l*-Al_2O_3 at 2127°C[1]. The first peak at *Q*=2.1 $Å^{-1}$ in X-ray and neutron *S*(*Q*) of *l*-Al_2O_3 and the second peak at *Q*=2.7 $Å^{-1}$ in neutron *S*(*Q*) can be assigned to the FSDP and the principal peak of Salmon *et al*[3], respectively.

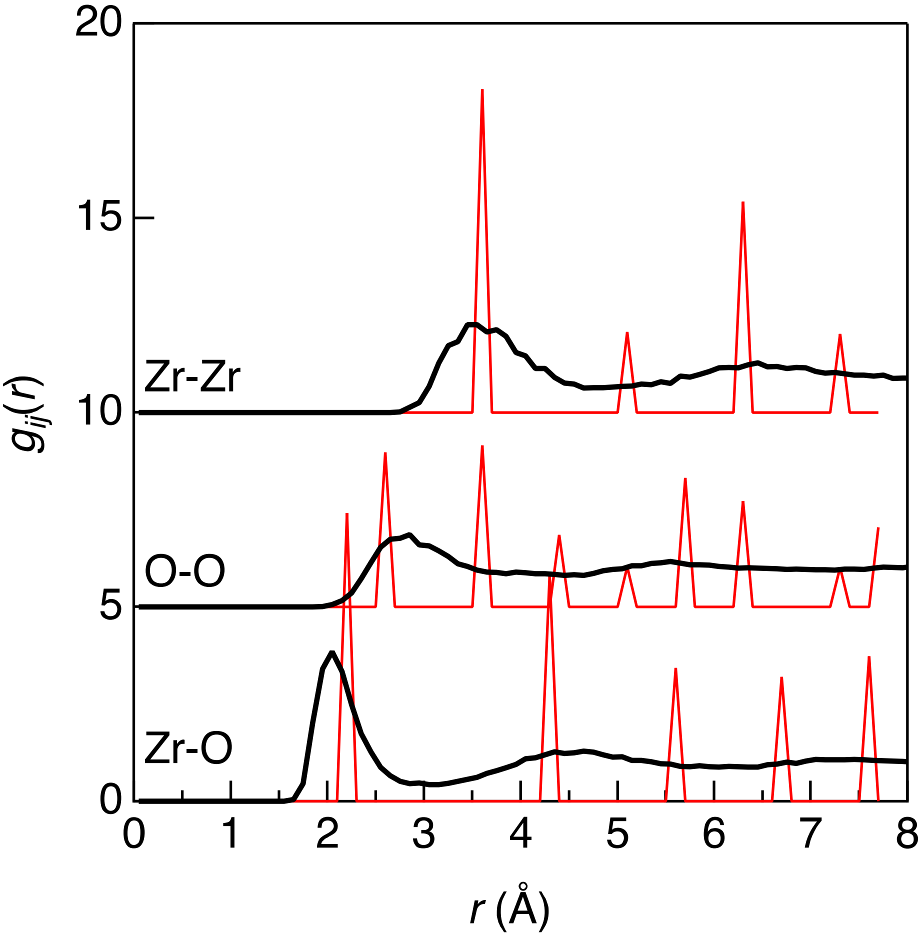

Supplementary Figure 4 | Faber-Ziman partial pair correlation functions, $g_{ij}(r)$, for *l*-ZrO_2 at 2800°C (black) and for the high-temperature phase of crystal (*c*-) ZrO_2 (red). The $g_{ij}(r)$ data of *c*-ZrO_2 were scaled by a factor of 3. *c*-ZrO_2 has ZrO_8 as a short-range structural unit[4], and the difference between crystal and liquid can clearly be seen in $g_{ZrO}(r)$ and $g_{OO}(r)$.

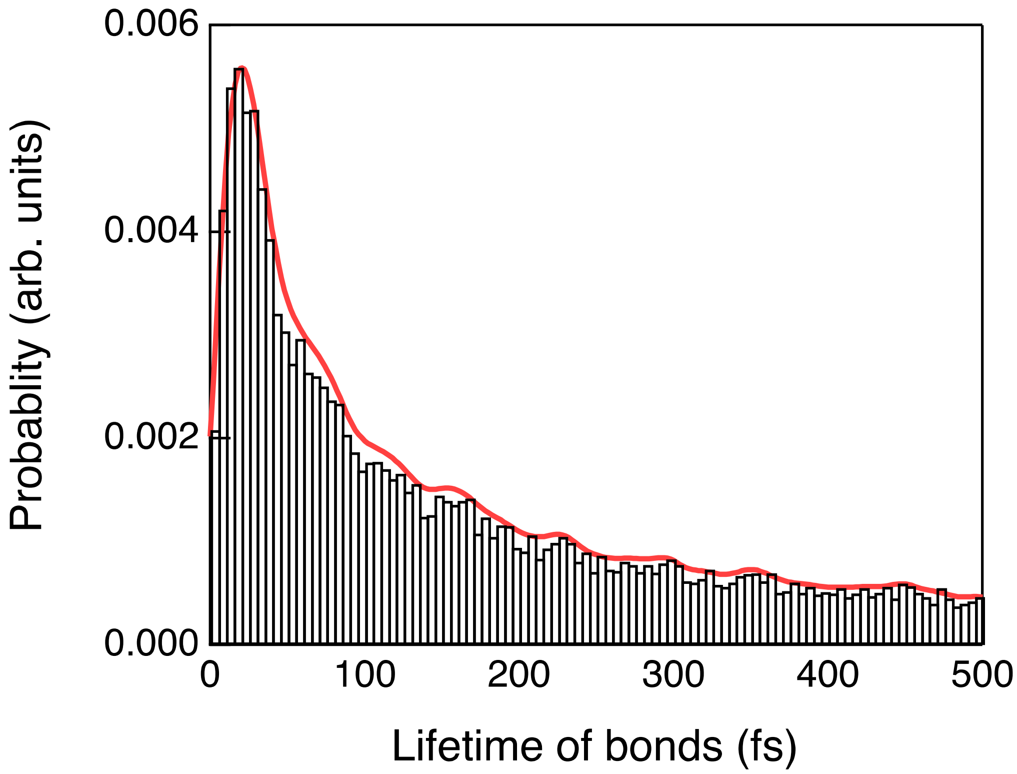

Supplementary Figure 5 | Lifetime of Zr-O bonds for *l*-ZrO_2 at 2800˚C.

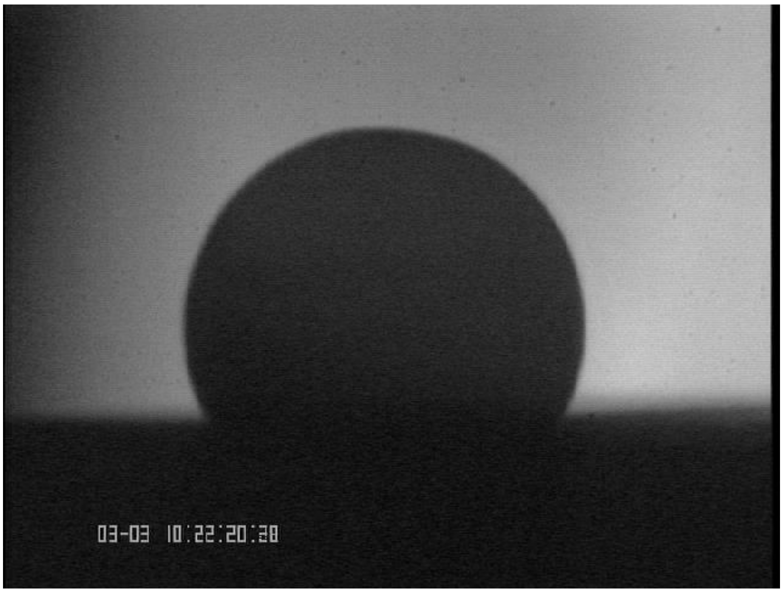

Supplementary Figure 6 | Magnified image of aerodynamically levitated *l*-ZrO_2.

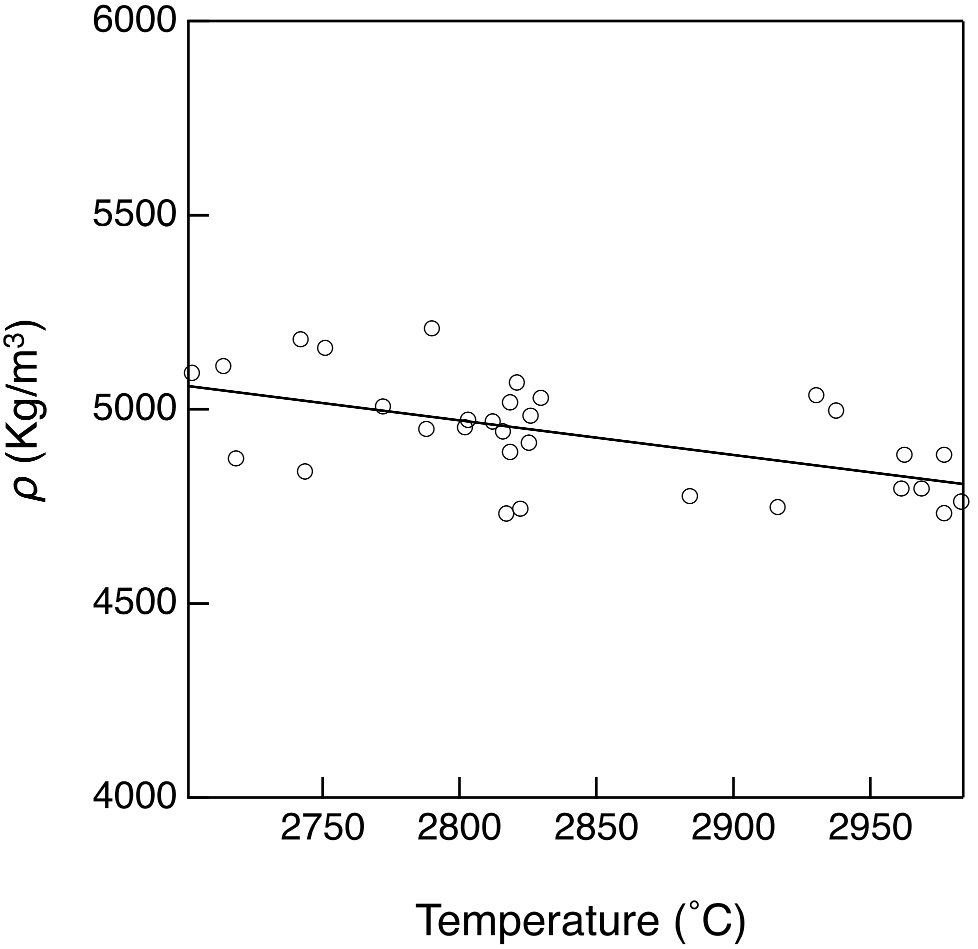

Supplementary Figure 7 | Measured density of *l*-ZrO_2 as a function of temperature. All data have error bars of 5 %.

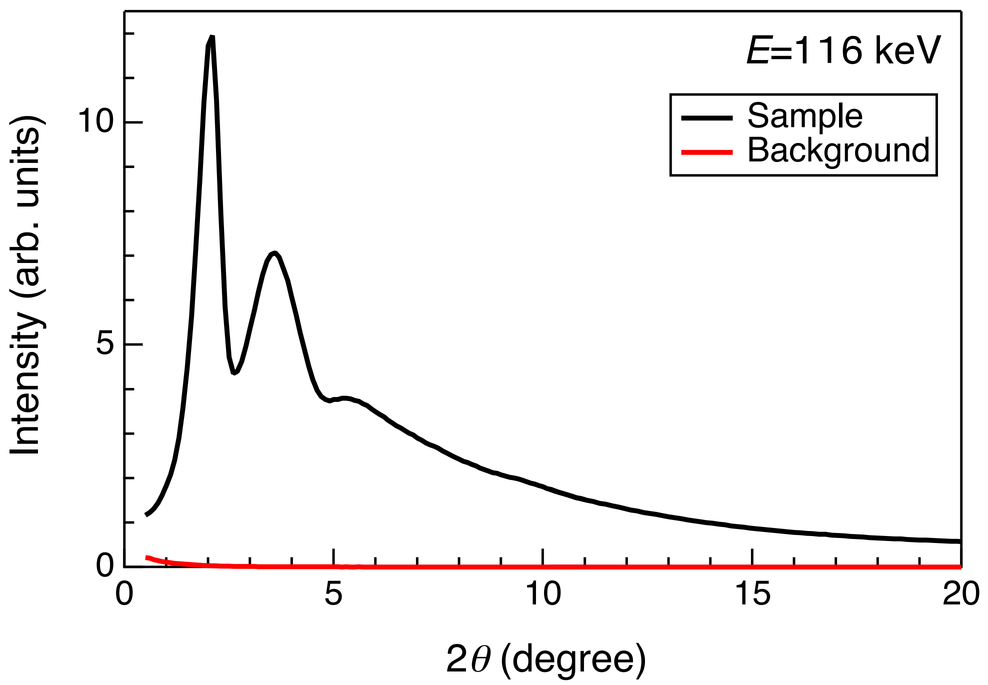

Supplementary Figure 8 | X-ray diffraction data for *l*-ZrO_2 at 2800˚C measured at the BL08W beamline of SPring-8.

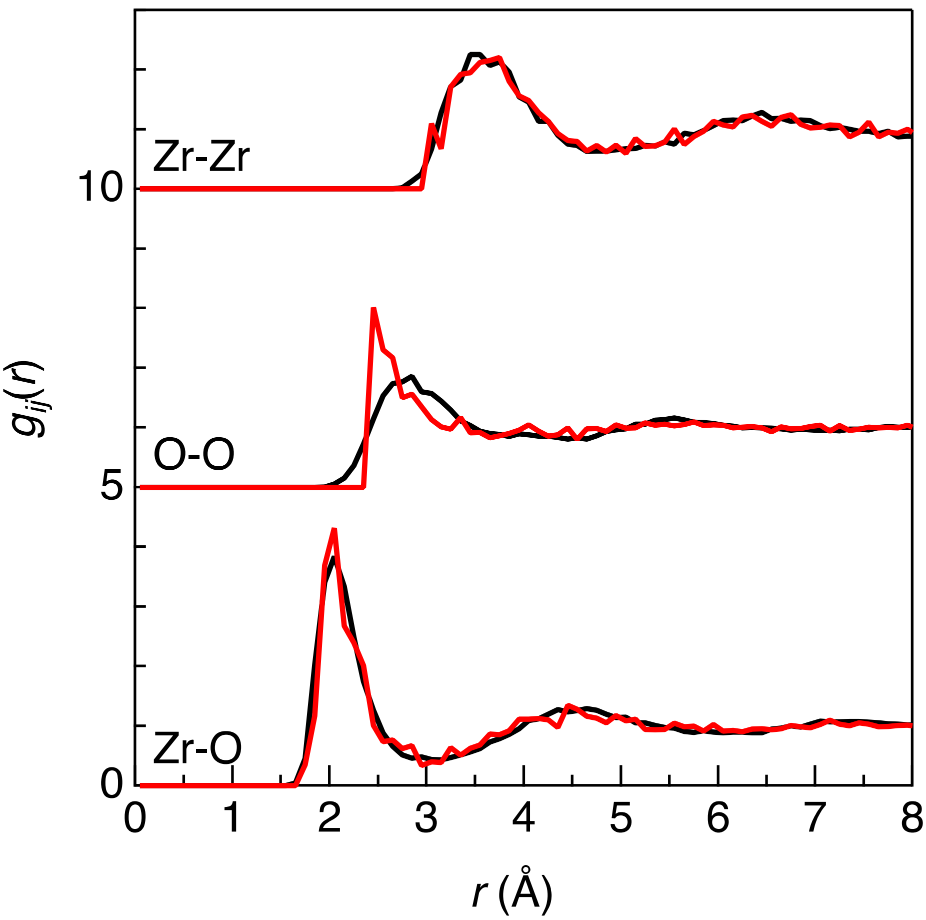

Supplementary Figure 9 | Faber-Ziman partial pair correlation functions, $g_{ij}(r)$, for *l*-ZrO_2 at 2800˚C from RMC modelling (red) and DF-MD simulation (black). The O-O $g_{ij}(r)$ in the RMC configuration (start) is artificially sharp due to small weighting factor for X-rays, while it is reasonable in the DF-MD simulation. The system loses its memory of the initial (RMC) starting structure within a few picoseconds (Zr-O bond lifetimes ~185 fs).

Supplementary Table 1 | Atomic charges and volumes of Zr cations and O anions in the high-temperature phase of *c*-ZrO_2 and *l*-ZrO_2 (Bader and Voronoi methods).

	Zr				O			
	Q_{eff} (e)		V_{at} (Å^3)		Q_{eff} (e)		V_{at} (Å^3)	
	Bader	Voronoi	Bader	Voronoi	Bader	Voronoi	Bader	Voronoi
c-ZrO_2	2.60	2.76	10.54	9.55	-1.30	-1.38	11.65	12.15
l-ZrO_2	2.62	2.40	11.48	12.48	-1.31	-1.20	14.72	13.48

Supplementary note 1 | Bhatia-Thornton partial structure factor $S_{\alpha\beta}(Q)$

The Bhatia-Thornton[5] number-number (N-N), concentration-concentration (C-C) and number-concentration (N-C) partial structure factors, denoted by $S_{NN}(Q)$, $S_{CC}(Q)$ and $S_{NC}(Q)$, respectively, were derived from the Faber-Ziman[6] partial structure factors $S_{ij}(Q)$. These structure factors separate the number density from the concentration fluctuations, and complement the information on topological order [$S_{NN}(Q)$] and chemical order [$S_{CC}(Q)$][3]. $S_{NC}(Q)$ describes the correlation between the number and concentration fluctuations.

Supplementary note 2 | Calculation of the lifetime of Zr-O bonds

The lifetimes were analyzed by tracking the Zr-O bonds during the density functional (DF) - molecular dynamics (MD) simulation. The algorithm checks all Zr-O bonds for each time step and stores lifetimes until bonds break (whenever the bond length exceeds the cutoff of 2.8 Å). New bonds and their lifetimes are also determined in the same way. The DF-MD simulations lasted 10 ps with a time step of 1 fs, and the analysis showed that 50% of the Zr-O bonds break within 185 fs at 2800°C.

Supplementary note 3 | Density measurement of liquid ZrO_2

The density measurement of liquid (*l*-) ZrO_2 was performed with an aerodynamic levitator. A small ZrO_2 sample of diameter around 2 mm was set in a shallow nozzle and levitated aerodynamically. The levitated sample was then heated by a 100 W CO_2 laser and a 500 W Nd:YAG laser. The temperature of the sample was measured by a single color pyrometer. The temperature was calibrated using the melting temperature (T_m: 2715 ˚C).

Sample imaging for density determination was achieved by a high-resolution, black-and-white CCD video camera (Sony SSC-M370) equipped with a telephoto objective and a high-pass filter (450 nm), in conjunction with a high-intensity ultraviolet background light. This allowed the perimeter to be analyzed[7]. The use of the ultraviolet lamp and the filter gave a background lighting efficiency that was practically independent of the sample temperature (from an overheated liquid to a room-temperature sample), thus allowing accurate determination the density. The image was analyzed during cooling, and the volume of the sample was calculated assuming that it was axially symmetric.. Details of the image analysis can be found elsewhere[8]. Since the bottom part of the sample is hidden by the nozzle, there is some uncertainty in this analysis. The density was calibrated by measuring a sample of liquid Al_2O_3 whose density had been measured by other methods[9]. The total uncertainty is up to 5 %. Supplementary Figure 6 shows the measured density as a function of temperature. When the measured data were fit to a linear function, the density (ρ) of *l*-ZrO_2 was given by

$$\rho = 5048 - 0.89\ (T\text{-}T_m),\ \mathrm{kg/m^3}. \qquad (1)$$

Supplementary References

[1]Skinner, L. B. *et al*. Joint diffraction and modeling approach to the structure of liquid alumina. *Phys. Rev. B* **87**, 024201 (2013).

[2]Takada, A., Richet, P., Catlow, C. R. A. & Price, G. D. Molecular dynamics simulations of vitreous silica structures. *J. Non-Cryst. Solids* **345&346**, 224–229 (2004).

[3]Salmon, P. S., Martin, R. A., Mason, P. E. & Cuello, G. J. Topological versus chemical ordering in network glasses at intermediate and extended length scales. *Nature* **435**, 75–78 (2005).

[4]Tomaszewski, P. E. Structural phase transitions in crystals. I. Database. *Phase Transition* **38**, 127–220 (1992).

[5]Bhatia, A. B. & Thornton, D. E. Structural aspects of the electrical resistivity of binary alloys. *Phys. Rev. B* **4**, 3004–3012 (1971).

[6]Faber, T. E. & Ziman, J. M. A theory of the electrical properties of liquid metals. *Philos. Mag.* **11**, 153–173 (1965).

[7]Ishikawa, T., Paradis, P.-F. & Yoda, S. New sample levitation initiation and imaging techniques for the processing of refractory metals with an electrostatic levitator furnace. *Rev. Sci. Instrum.* **72**, 2490–2495 (2001).

[8]Chung, S.-K., Thiessen, D. B. & Rhim, W.-K. A noncontact measurement technique for the density and thermal expansion coefficient of solid and liquid materials. *Rev. Sci. Instrum.* **67**, 3175–3181 (1996).

[9]Paradis, P.-F., Ishikawa, T., Saita, Y., & Yoda, S. Non-contact thermophysical property measurements of liquid and undercooled alumina. *Jpn. J. Appl. Phys.* **43**, 1496–1500 (2004).
